# HspB5 Activates a Neuroprotective Glial Cell Response in Experimental Tauopathy

**DOI:** 10.3389/fnins.2020.00574

**Published:** 2020-06-11

**Authors:** David W. Hampton, Sandra Amor, David Story, Megan Torvell, Malika Bsibsi, Johannes M. van Noort, Siddarthan Chandran

**Affiliations:** ^1^Centre for Clinical Brain Sciences, The University of Edinburgh, Edinburgh, United Kingdom; ^2^Department of Pathology, VU University Medical Center, Amsterdam, Netherlands; ^3^UK Dementia Research Institute, Cardiff University, Cardiff, United Kingdom; ^4^Delta Crystallon, Leiden, Netherlands; ^5^UK Dementia Research Institute, Edinburgh University, Edinburgh, United Kingdom

**Keywords:** heat shock protein, astrocytes, microglia, tau, neurodegeneration, neuroprotection

## Abstract

Progressive neuronal death during tauopathies is associated with aggregation of modified, truncated or mutant forms of tau protein. Such aggregates are neurotoxic, promote spreading of tau aggregation, and trigger release of pro-inflammatory factors by glial cells. Counteracting such pathogenic effects of tau by simultaneously inhibiting protein aggregation as well as pro-inflammatory glial cell responses would be of significant therapeutic interest. Here, we examined the use of the small heat-shock protein HspB5 for this purpose. As a molecular chaperone, HspB5 counteracts aggregation of a wide range of abnormal proteins. As a TLR2 agonist, it selectively activates protective responses by CD14-expressing myeloid cells including microglia. We show that intracerebral infusion of HspB5 in transgenic mice with selective neuronal expression of mutant human P301S tau has significant neuroprotective effects in the superficial, frontal cortical layers. Underlying these effects at least in part, HspB5 induces several potent neuroprotective mediators in both astrocytes and microglia including neurotrophic factors and increased potential for removal of glutamate. Together, these findings highlight the potentially broad therapeutic potential of HspB5 in neurodegenerative proteinopathies.

## Introduction

Accumulation of hyperphosphorylated and aggregated tau is a hallmark of neurodegenerative diseases such as Alzheimer’s disease and other tauopathies (Spillantini and Goedert, 2013). Mutations in tau cause inherited frontotemporal dementia (FTD) establishing that abnormality of tau protein alone is sufficient to cause neurodegeneration (Lee et al., 2001). Overexpression of human mutant P301S tau in mice under a neuron-specific promoter has previously been shown to recapitulate the core features of tauopathies including progressive neuronal loss, a neuroinflammatory response and accumulation of hyperphosphorylated tau, thus making this model suitable for the evaluation of putative therapeutic strategies (Bellucci et al., 2004, 2011; Sasaki et al., 2008; Hampton et al., 2010; Jucker and Walker, 2011; Holmes et al., 2014; Torvell et al., 2019).

As a molecular chaperone, HspB5 counteracts aggregation, eliminates neurotoxicity of a wide range of different abnormal protein aggregates (Waudby et al., 2010; Mannini et al., 2012; Yerbury et al., 2013; Hochberg et al., 2014) and inhibits hyperphosphorylation of tau and its aggregation into oligomers and insoluble fibers *in vivo* (Dabir et al., 2004; Björkdahl et al., 2008). In experimental neurodegenerative diseases, e.g., Alzheimer’s and Parkinson’s, increased expression of HspB5 in neural cells correlates with reduced damage and improved functional recovery (Ojha et al., 2011; Marino et al., 2015). Evidence from the neuroinflammation field has revealed the additional ability of HspB5 to activate a TLR2-mediated neuroprotective and anti-inflammatory response in CD14-expressing myeloid cells including microglia (van Noort et al., 2010, 2013; Bsibsi et al., 2013, 2014). In line with these biological activities, administration of HspB5 ameliorates neuroinflammation, stroke, spinal cord injury, and optic nerve damage in experimental models (Ousman et al., 2007; Arac et al., 2011; Pangratz-Fuehrer et al., 2011; Klopstein et al., 2012; Wu et al., 2014), and symptoms of multiple sclerosis in humans (van Noort et al., 2015). In the present study, we examined its effects in an experimental model of human tauopathy. Upon intracerebral infusion in P301S mice, HspB5 led to a significant neuroprotective effect, which was associated with protective responses by both microglia and astrocytes.

## Results and Discussion

### HspB5 Is Neuroprotective in an *in vivo* Model of Tauopathy

**FIGURE 1:**
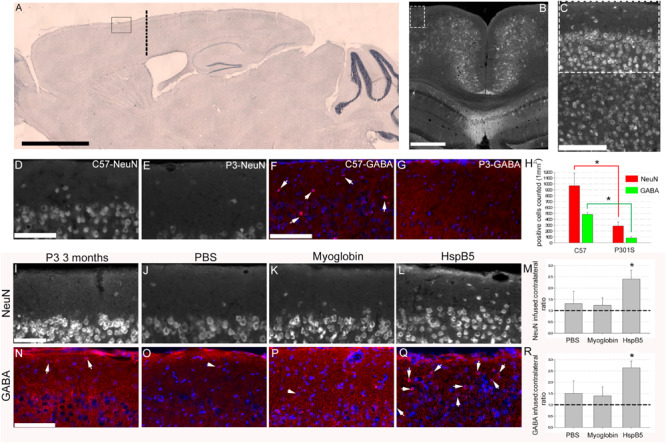
Intracerebral infusion of HspB5 is neuroprotective in P301S mice. Overview images **(A–C)** to show where canula were inserted (co-ordinates based on Bregma detailed in methods) as well as area of analysis. Sagittal image from a C57/BL6 stained with cresyl violet providing an overview of infusion site based on cannulas being inserted through the skull to a depth of 0.5 mm to touch the surface of the cortex, 0.75 mm lateral from the midline and 1.2 mm forward from bregma, marked by the thick dotted black line with the box highlighting area for analysis **(A)**, scale bar = 2 mm. Coronal image highlighting a potential area of analysis in the dotted box, scale bar = 500 μm with **(C)** showing this area zoomed in and the dotted box in **(C)** showing the representative area where all following analysis would be performed in, scale bar = 100 μm. P301S (*n* = 4) mice compared to age-matched C57/BL6 (*n* = 7) control mice **(D–H)**. Scale Bars = 100 μm for **(D–G)** and quantification of both NeuN and GABAergic interneurons within this superficial region **(H)** (asterisks signifying *P* < 0.05, one way ANOVA, Tukey test) in the P301S mice when compared to the C57/BL6 mice. P301S mice (at 8 weeks old) were either not treated (**I,N**, *n* = 4), or infused with PBS (**J,O**, *n* = 5) myoglobin (**K,P**, *n* = 7), or HspB5 (**L,Q**, *n* = 10) in a continuous manner using osmotic mini-pumps connected to a canula overlaying the superficial surface of the cortex for 4 weeks. At 12 weeks of age, the superficial cortex was examined for viable neurons expressing either NeuN **(I–M)** or GABA (**N–R**; arrows highlight GABA+ neurons). **(M,R)** Show quantification of the numbers of viable neurons relative to the untreated contralateral hemisphere, with the dotted line signifying normal 12 weeks old P301S mice(asterisk signifying *P* < 0.01, one way ANOVA, Tukey test). All scale bars are shown for grouped images therefore panels **(I–L,N–Q)** the scale is the same with a representative bar shown in **(I)** or **(N)** = 100 μm.

Here, we show that implantation of a cannula (at 8 weeks of age, location and area analyzed in the superficial motor cortex shown in Figures 1A–C) and infusing PBS or myoglobin for 4 weeks did not alter the loss of NeuN+ or GABAergic+ neurons (Figures 1I–R). This is important as we have reported previously (Hampton et al., 2010; Torvell et al., 2019) that numbers of NeuN+ and GABA+ neurons in the superficial frontal cortex are significantly decreased in 12-week old P301S mice as compared to 8-week P301S mice or healthy, 12-week old C57/BL6 mice. Thus validating this observation again here we show that NeuN+ (969.7 ± 211.3 for C57/BL6 compared to 285.6 ± 71.5 in P301S) and GABA+ (482.6 ± 34.3 for C57/BL6 compared to 83.3 ± 23.5 in P301S) neurons are lost in this superficial frontal motor cortex by 12 weeks of age (Figures 1D–H). Also numbers of GFAP+ reactive astrocytes are increased, thus further verifying this age-dependent model of tau-driven, superficial frontal cortical neuronal loss. To normalize data a ratio of the contralateral, non-infused hemisphere to infused hemisphere was generated. This revealed a ratio of 1.3 ± 0.5 and 1.2 ± 0.3 for PBS and myoglobin infusion respectively thus confirming that control infusions (be it either PBS or myoglobin) had no impact on neuronal survival (Figures 1M,R). In contrast, neuronal density ratio was significantly increased following 4-week infusion of HspB5 (2.4 ± 0.4 for NeuN+; 2.6 ± 0.3 GABA+ neurons) (Figures 1L,M,Q,R), similar to 12-week old C57/BL6 control mice, or P301S mice prior to neuronal loss. Thus, infusion with HspB5 is neuroprotective, significantly preventing neuronal loss in the outer cortical layers.

### HspB5 and Glial Activation in an *in vivo* Model of Tauopathy

**FIGURE 2:**
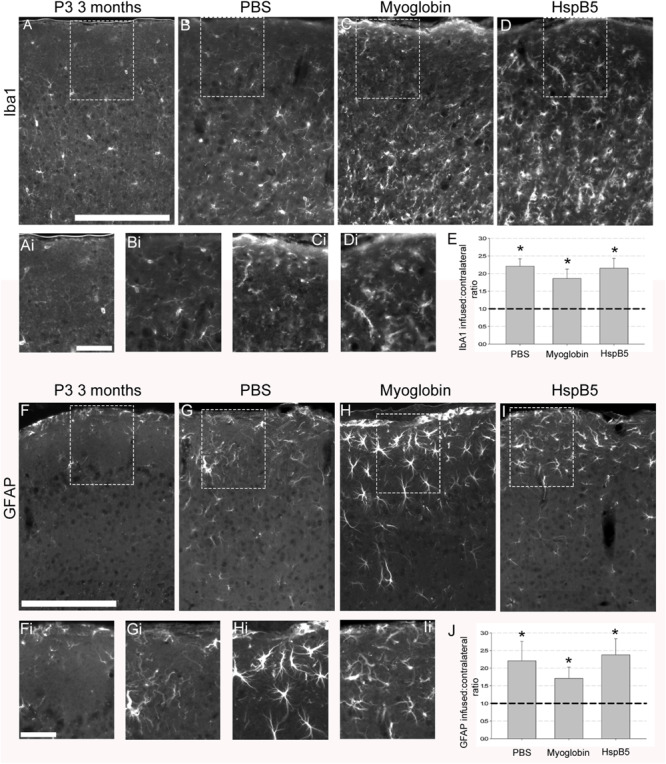
Increase in reactive glial markers following infusion of PBS, myoglobin or HspB5. P301S mice (8 weeks old) were infused with PBS (*n* = 5), myoglobin (*n* = 7), or HspB5 (*n* = 10) for 4 weeks and the superficial cortex was examined in identical areas as the neuronal analysis for Iba1 expression (pan-microglial marker, **A–D**) or GFAP (reactive astrocytes, **F–I**). The dotted boxes in panels **(A–D,F–I)** have zoomed in inserts as marked by the addition of a ‘i’ to their respective panels, i.e., **(Ai)** is the boxed area from **(A)**. Quantification of the ratio of glial markers **(E,J)**, with the asterisks *, signifying *P* < 0.01 in a one Way ANOVA, Tukey test. All scale bars are shown for grouped images therefore panels **(A–Ii)** the scale is the same with a representative bar shown in **(A,F)** = 200 μm, **(Ai,Fi)** = 50 μm.

Next, the impact of the infusions on numbers of Iba-1+ microglia and GFAP+ reactive astrocytes were examined. As shown in Figures 2A–J, infusions of PBS or myoglobin or HspB5 all induced a significant increase in the number of reactive glial cells, with no differences between each infused group except when compared to normal 12 weeks old P301S mice. Thus PBS (2.2 ± 0.2 Iba1; 2.2 ± 0.5 GFAP), myoglobin (1.9 ± 0.3 Iba1 and 1.7 ± 0.3 GFAP) and HspB5 (2.2 ± 0.3 Iba1; 2.4 ± 0.4 GFAP) all increased these reactive glial markers to a similar extent. Therefore it was not an increase in the numbers of reactive glia that was responsible for the observed neuroprotective effect of HspB5 only, leading us to further examine the impact of HspB5 on glial phenotype.

### HspB5 Induces a Secondary Neuroprotective Response in Astrocytes

**FIGURE 3:**
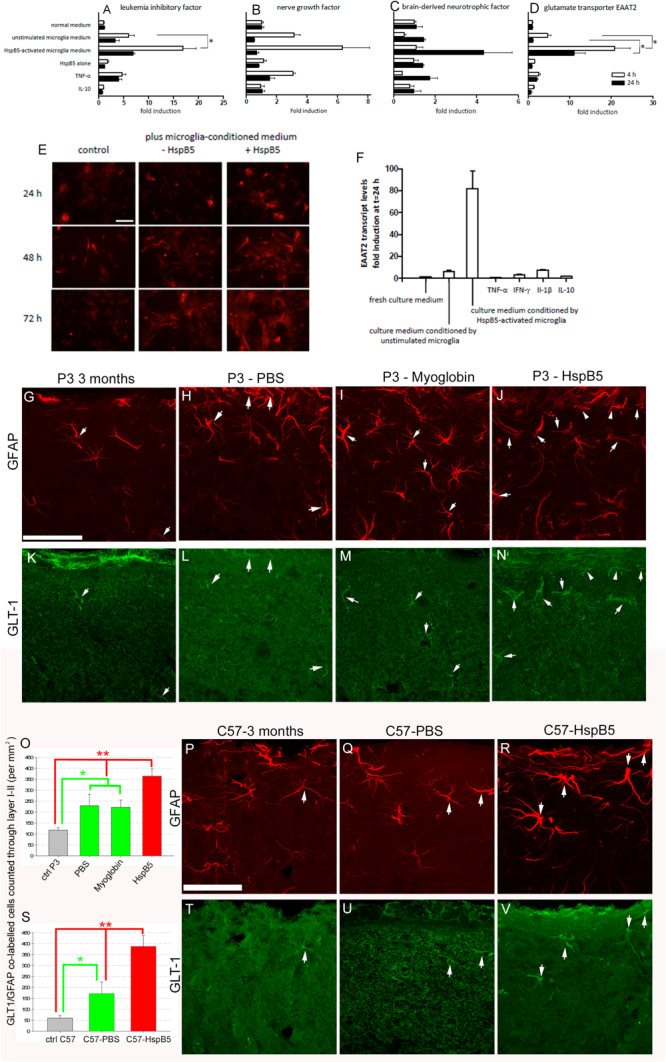
Neuroprotective astrocyte responses to HspB5 depend on concomitant exposure to soluble factors from HspB5-activated microglia. Cultured human adult astrocytes were exposed to different stimuli including HspB5 alone or in the context of microglia-conditioned media, and transcript levels of LIF **(A)**, NGF **(B)**, BDNF **(C)**, and EAAT2 **(D)** were determined after 4 and 24 h. In a separate set of experiments, EAAT2 protein expression on cultured human astrocytes was examined by immunocytochemical staining at various points in time after stimulation with HspB5 either alone or in the context of microglia-conditioned medium **(E)**, scale bar = 10 μm for all image panels. In parallel, EAAT2-encoding transcripts were again quantified by RT-PCR **(F)** ^∗^*P* < 0.01. Lower panels **(G–N)** illustrate *in vivo* expression of GLT-1 by GFAP+ astrocytes following intracerebral infusion of P301S mice with PBS (*n* = 5), myoglobin (*n* = 7), or HspB5 (*n* = 10). The white arrows highlight examples of GFAP+ and GLT1+ co-labeling with panel **(O)** providing quantification of this expression, with differences evaluated by two-way ANOVA, Tukey, *P < 0.05; **P < 0.01. **(P–R,T–V)** Are representative images from C57/Bl6 mice also infused with either PBS (*n* = 3), myoglobin (*n* = 4), or HspB5 (*n* = 5) and again panel **(S)** provides quantitation of this expression, with differences evaluated by two-way ANOVA, Tukey, *P < 0.05; **P < 0.01. All scale bars are shown for grouped images therefore panels **(G–N,P–V)** the scale is the same with a representative bar shown in **(G,P)** respectively, scale bar = 50 μm.

Previously we have shown that transplantation of astrocytes can be neuroprotective in the P301S mouse (Hampton et al., 2010). Given that activation of innate cellular responses by HspB5 is mediated via TLR2, with CD14 as an essential co-receptor (van Noort et al., 2010; Bsibsi et al., 2013, 2014), which are absent on astrocytes, any direct activation of astrocytes by HspB5 would be unexpected. Indeed, investigations of treatment of cultured human astrocytes with HspB5 showed no significant increase in neurotrophic factors including NGF and BDNF previously shown to be increased following astrocyte transplantation into the P301S mice (Hampton et al., 2010) (Figures 3A–C). However, exposure to HspB5 accompanied by soluble factors released by HspB5-activated human microglia did trigger astrocyte activation of BDNF, NGF, and LIF (Figures 3A–C) and resulted in significantly increased production of EAAT2 (Figure 3D). EAAT2, the human equivalent of GLT-1, is an astrocyte specific glutamate reuptake transporter that is critical to regulating glutamate homeostasis at the synaptic cleft (Soni et al., 2014). Expression of EAAT2 on HspB5-activated astrocytes was also confirmed by immunocytochemical staining (Figure 3E) and quantification of EAAT2 transcripts showed an 80-fold increase (Figure 3F). Individual pro-inflammatory mediators (including TNFα or interleukin-10) alone or culture medium from unstimulated microglia did not increase EAAT2 production on astrocytes. Thus, the combination of HspB5 with products secreted by HspB5-activated microglia produced this significant induction in astrocytes of EAAT2 and other neuroprotective factors (Figures 3A–F). In support of these *in vitro* findings, analysis of HspB5-treated P301S mouse brains revealed a significant increase in GLT-1 positive/GFAP positive astrocytes (364 ± 32.8 per mm^2^) compared to myoglobin (221.9 ± 33.1 per mm^2^) or PBS (229.3 ± 51.3) not only in P301S mice (Figures 3G–O) but also C57/BL6 control animals (Figures 3P–V).

Our present findings demonstrate that the significant neuroprotective effects of HspB5 in the P301S model of tauopathy are associated with activation of local glial cells. Previous data have already documented induction in microglia of several anti-inflammatory and neuroprotective mediators by HspB5 (van Noort et al., 2010; Bsibsi et al., 2013, 2014). Our current findings extend this by revealing that astrocytes can also respond to HspB5 in a neuroprotective manner, but only when simultaneously exposed to the secreted products of HspB5-activated microglia as cofactors. Together, these data provide the first experimental evidence of a neuroprotective effect of HspB5 in a model of inherited neurodegeneration due to mutant tau.

## Materials and Methods

### Treatment of P301S Mice

P301S transgenic mice (female, 8 weeks age) were anesthetized and positioned into a stereotaxic frame (David Kopf Instruments, Tujunga, CA, United States). Alzet micro-osmotic pumps model 1004 (Durect Corporation, Cupertino, CA, United States) were filled with 100 μl of a sterile solution of PBS containing 12.5 mg/mL recombinant human HspB5 (Delta Crystallon, Leiden, NL), 12.5 mg/mL myoglobin (M5696; Sigma Aldrich, St. Louis, MO, United States), or PBS-only resulting in infusions of an average of 33 μg of total protein, within roughly 3 μl of solution per day at a rate of 0.1 μl per hour, via a cannula (Alzet brain infusion kit 3) positioned approximately 0.5 mm deep, resting onto the surface of the cortex 0.75 mm laterally from the midline and 1.2 mm forward from bregma as shown in Figure 1. This area was chosen for the infusions as we have previously shown significant neuronal loss within this region of the motor cortex at 12 weeks of age in P301S mice.

Animals were euthanized 4 weeks post-cannulation at 12 weeks of age, perfused with 4% paraformaldehyde in PBS. Brains were removed, post-fixed in 4% paraformaldehyde overnight, cryoprotected in 25% sucrose solution, and frozen in tissue-tec. Cryosections were cut coronally (25 μm), mounted onto superfrost-plus glass slides (VWR International, East Grinstead, United Kingdom) and stored at −80°C. Sections were blocked in 3% serum solution (goat, sheep or horse serum chosen depending on secondary antibodies to be used) in a 0.2% triton-X PBS solution for 1 h before being removed and primary antibodies added (Table 1) in a 0.2% triton-X PBS solution containing 1% of the appropriate serum for blocking. After washing in PBS, secondary antibodies (Table 1) were applied. Slides were washed in PBS and mounted using Fluorsave reagent (Calbiochem, Nottingham, United Kingdom). These methods have been detailed previously (Hampton et al., 2004, 2007, 2010; Torvell et al., 2019).

**TABLE 1:** Antibodies.

**Catalog #**	**Antigen/target**	**Source**	**Species generated**	**Dilution**
**Primary Antibodies**				
MAB377	NeuN-biotinylated	Millipore Bioscience	Mouse monoclonal	1:400
A2052	GABA	Sigma-Aldrich	Rabbit polyclonal	1:500
ab5076	IBA1	Abcam	Goat polyclonal	1:150
C9205	GFAP-Cy3 conjugated	Sigma-Aldrich	Mouse monoclonal	1:400
GLT11-A	GLT-1	Alpha-Diagnostic International	Rabbit polyclonal	1:200
MCA839	PLP	Ab D serotec	Mouse monoclonal	1:500
14-9956-82	HLA-DR (LN3)	e-bioscience	Mouse monoclonal	1:500
MON-RTU1113	GFAP	Monosan	Mouse monoclonal	1:50
−	PTX3	In house	Rabbit	1:1000
GA524	GFAP	Dako	Rabbit polyclonal	1:3000
011-27991	Iba-1	Wako	Rabbit polyclonal	1:10000
**Secondary Antibodies**				
SK11021-2	Envision	DAKO	Goat	Ready to use
A-11005	IgG-594	Invitrogen	Goat anti mouse	1:250
A-11008	IgG-488	Invitrogen	Goat anti rabbit	1:250
4030-04	AP	Southern biotech	Goat anti rabbit	1:250
S11226	Streptavidin – 568	Molecular probes/Invitrogen	–	1:1000
A-11034	IgG Alexa-488	Molecular probes/Invitrogen	Goat anti rabbit	1:1000
A-11055	IgG Alexa-488	Molecular probes/Invitrogen	Donkey anti goat	1:1000

### Analysis of Neurons and Glia From *in vivo* Experiments

Axiovision 4.8 (Zeiss microsystem, Cambridge, United Kingdom) was used to collect images on a Zeiss Axiovision upright microscope or Apotome system, or Zen 2009 software was used in conjunction with a Zeiss LSMZ10 confocal microscope. Images were taken as z-stacks and max-projected prior to analysis. All images within a dataset were captured and handled identically for quantitative analysis. Zen blue or Axiovision (Zeiss) or SigmaScan Pro 5.0 (SPSS, Chicago, IL, United States) software were used for subsequent quantitative density measurements and cell counts.

For initial verification analysis of 12 weeks old P301S (*n* = 4) compared to age-matched C57/BL6 (*n* = 7) NeuN and GABA positive cells were counted in a 155 × 155-μm grid throughout the superficial cortex, using 4 images per animal, with all cellular counts expressed as mean numbers of cells per 1 mm^2^ of tissue (Figure 1E).

For analysis of NeuN (Figure 1) and GFAP (Figure 2) expression, images were taken throughout the superficial cortex and converted to grayscale max-projection TIFFs. All images were then thresholded such that pixels overlying immune-positive cells had a grayscale value of 68, and all other pixels had a value of 0. Therefore, an average intensity could be converted to density measurements by dividing the output by 68, leading to a scale of 0 (minimum) to 1 (maximum possible reading) being generated. These thresholded images were then use to generate a percentage area of the cortex containing a positive antibody signal, by dividing the number of positive readings by the total number of potential readings, multiplied by 100. Between 6 and 8 sections per animal were analyzed, from which a mean percentage area of the cortical layer containing a positive NeuN or GFAP signal could be calculated. For analysis of GABA+ neurons (Figure 1) and IbA1+ microglia (Figure 2) cells were counted in a 155 × 155-μm grid throughout the superficial cortex, using between 6 and 8 images per animal. To be able to compare multiple experiments across different times and institutes, each group of data except GFAP-GLT1 analysis was normalized to generate a ratio of infused hemisphere to normal contralateral hemisphere before being unblinded and grouped correctly.

For GFAP+ GLT-1+ double-labeled positive astrocytes (Figure 2) cells were counted in a 155 × 155-μm grid throughout the superficial cortex, using between 6 and 8 images per animal, with all cellular counts being expressed as mean numbers of cells per 1 mm^2^ of tissue.

N numbers for the various groups are shown in their relevant figure legends.

### Isolation and Culturing of Human Microglia, Astrocytes and Macrophages

Human adult microglia and astrocytes were isolated from fresh post-mortem brain samples as previously described (Dabir et al., 2004; Björkdahl et al., 2008). Conditioned media was generated by supplying microglia with fresh culture medium containing 50 μg/mL HspB5 and medium harvested after 24 h. Microglia-conditioned media was used for astrocyte stimulations at a 1:1 dilution in the astrocyte culture medium. Astrocyte stimulations with control stimuli were performed by addition of recombinant human TNF-α, IFN-γ IL-1β or IL-10 (PeproTech Inc), all at a final concentration of 2 ng/mL. For RT-PCR analyses of transcripts at 4 or 24 h of stimulation, total cellular RNA was isolated from astrocytes using TRizol^®^ according to the manufacturer’s protocol (Invitrogen, Breda, Netherlands). RNA was reverse transcribed into cDNA and transcripts levels relative to β-actin were determined by RT-PCR using SYBR^®^ green.

The primers used (Biolegio, Nijmegen, Netherlands) were the following: EAAT-2 forward: TTCCCTGAAAACCTTGTCCA and reverse: GGTGGTGCAACCAGGACTTT; LIF forward: CC AACGTGACGGACTTCCC and reverse: TACACGACTATGCG GTACAGC; BDNF forward: CTACGAGACCAAGTGCAATCC and reverse: AATCGCCAGCCAATTCTCTTT; NGF forward: GGCAGACCCGCAACATTACT and reverse: CACCACCGAC CTCGAAGTC; β-actin forward: GGTCATCACCATTGGCAA and reverse: ACGTCACACTTCATGATG.

For immunocytochemical staining of EAAT2, human astrocytes cultured in chamber slides were rinsed with PBS and fixed for 10 min at room temperature with 4% (w/v) paraformaldehyde. To permeabilize the cells and block non-specific protein-protein interactions, astrocytes were incubated in 1% (w/v) BSA/10% (v/v) normal goat serum/0.3 M glycine in 0.1% (v/v) Tween in PBS for 1 h. Staining was performed by incubation of cells overnight at 4°C with 5 μg/ml rabbit polyclonal anti-EAAT2 antibody (ab41621, Abcam) diluted in 5% (v/v) normal goat serum. Cells were next washed in PBS and incubated for 1 h with goat anti-rabbit Alexa fluor-594 (H + L) (Life Technologies).

### Statistics

Graphs were generated using graphing software (Sigma Plot 11) and statistical tests were performed using SigmaStat and differences analyzed for statistical significance with ANOVA and Tukey *post hoc* test and two-tailed Student’s *t*-test. *P*-values of less than 0.05 were considered statistically significant and whenever applied, all *P*-values are shown in the respective figure legends.

### Study Approval

Animal procedures complied with national and institutional guidelines (UK Animals Scientific Procedures Act 1986, University of Cambridge and Edinburgh Animal Care Committees) and adhered to the ARRIVE guidelines.

Human tissues were obtained via the rapid autopsy regimen of the Netherlands Brain Bank in Amsterdam (coordinator Dr. I. Huitinga), and with the approval of the medical ethical committee of the VUMC Amsterdam.

## Data Availability Statement

The original contributions presented in the study are included in the article/supplementary material, further inquiries can be directed to the corresponding author(s).

## Ethics Statement

Animal procedures complied with national and institutional guidelines (UK Animals Scientific Procedures Act 1986, University of Cambridge and Edinburgh Animal Care Committees) and adhered to the ARRIVE guidelines. Human tissues were obtained via the rapid autopsy regimen of the Netherlands Brain Bank in Amsterdam (coordinator Dr. I. Huitinga), and with the approval of the medical ethical committee of the VUMC Amsterdam. The animal study was reviewed and approved by the University of Edinburgh Animal Care Committee and the University of Cambridge Animal Care Committee.

## Author Contributions

DH, JN, and SC designed the study. DH, DS, MT, and MB performed the experiments. SA provided the human glia cultures. All authors contributed to the final manuscript.

## Conflict of Interest

The authors declare that the research was conducted in the absence of any commercial or financial relationships that could be construed as a potential conflict of interest.
